# The determinants of the changing speed of spread of COVID-19 across Italy

**DOI:** 10.1017/S095026882200084X

**Published:** 2022-05-06

**Authors:** Pierluigi Cocco, Sara De Matteis

**Affiliations:** 1 Division of Population Health, Centre for Occupational and Environmental Health, University of Manchester, Manchester M13 9PL, UK; 2 Department of Medical Sciences and Public Health, University of Cagliari, 09047 Monserrato, Italy

**Keywords:** COVID-19, doubling time, cumulative incidence, case fatality rate, epidemic, environmental epidemiology

## Abstract

The COVID-19 epidemic showed inter-regional differences in Italy. We used an ecological study design and publicly available data to compare the basic reproduction number (*R*_0_), the doubling time of the infection (DT) and the COVID-19 cumulative incidence (CI), death rate, case fatality rate (CFR) and time lag to slow down up to a 50-days doubling time in the first and the second 2020 epidemic waves (*δ*DT50) by region. We also explored socio-economic, environmental and lifestyle variables with multiple regression analysis. COVID-19 CI and CFR changed in opposite directions in the second *vs*. the first wave: the CI increased sixfold with no evidence of a relationship with the testing rate; the CFR decreased in the regions where it was initially higher but increased where it was lower. The *R*_0_ did not change; the initially mildly affected regions, but not those where the first wave had most severely hit, showed a greater *δ*DT_50_ amplitude. Vehicular traffic, average temperature, population density, average income, education and household size showed a correlation with COVID-19 outcomes. The deadly experience in the first epidemic wave and the varying preparedness of the local health systems might have contributed to the inter-regional differences in the second COVID-19 epidemic wave.

## Introduction

The swaying evolution of viral pandemics is well known [1], but the determinants of the period and amplitude of their oscillations and the geographical variation in spreading are not clearly understood. We explored what factors might have contributed using an ecological study design.

The first Italian case of COVID-19 was diagnosed on 20 February 2020. The following day, the diagnoses were 15 in Lombardy, three in Latium and two in Veneto. By 5 March, the infection had spread all over the 20 Italian regions. Between 8 and 21 March, the Italian Government issued a nationwide lockdown. On 11 and 21 March, two Prime Minister decrees closed all schools and public services but local transportation and hospitals; all intercity travel, social events and non-essential commercial activities were prohibited. Remote working was imposed on most public employees; wearing facial masks became mandatory indoors and outdoors. On 21 March, the incident cases reached the top (No = 6557); on 31 March, the count of deaths was the highest (No = 919) [2]. The restrictions were gradually eased in May.

According to the joint working group of the Italian National Institute of Statistics (ISTAT) and the National Institute of Health (Istituto Superiore di Sanità, ISS), the second phase of the COVID-19 epidemic started by the end of September 2020 [3]. Progressive restrictions took place between 8 October and 3 November when a nationwide night-time curfew was introduced. These included: shutting down social events, prohibiting mass gatherings and extending to outdoors the mandatory use of facial masks. Based on the changes in several parameters, on 6 December, the regions were classified into three zones of increasing restrictions: yellow (including Emilia Romagna, Friuli Venezia Giulia, Latium, Liguria, Marche, Molise, province of Trentino, Apulia, Sardinia, Sicily, Umbria and Veneto), orange (Basilicata, Calabria, Campania, Lombardy, Piedmont, autonomous province of Alto Adige, Tuscany and Aosta Valley) and red (Abruzzo). The top incident cases occurred on 13 November (No = 40 902); afterwards, the epidemic curve slowly declined up to 28 December (No = 9072), to increase again in the following days [2]. Most restrictions were lifted between 19 May and 4 June 2021.

A previous study described an inverse relationship of varying amplitude between cumulative incidence (CI) in the first *vs*. the second COVID-19 epidemic wave across the Italian provinces [4]. On the other hand, the age- and gender-standardised case fatality rate (CFR) was three times higher in the first epidemic wave. The origin of such striking differences would include the complex interplay between the viral evolution, the host resistance, environmental circumstances modulating the probability of contagion and the compliance of the local population with the control measures, such as wearing facial masks and maintaining the social distance. Although the evolutionary rate of SARS-CoV-2 appears to be relatively slow, several variants were identified in various parts of the world [5]. In 2020, two were mostly relevant globally: the first, identified in late January 2020 in China and Germany, carried the D614G mutation in the spike protein of the ancestral genome and was associated with a rapid spread worldwide, a high fatality rate and a high viral load [6–8]. In the last quarter of 2020, the *α* variant, first sequenced in Great Britain, took over. It included several mutations in the receptor-binding domain of the spike protein and was reportedly associated with an increase in transmissibility and mortality [5, 9]. However, as sequencing data of the viral genome was not uniformly available in 2020, the contribution of new variants in determining time and space changes in the epidemic curve is unclear. As it concerns the environmental circumstances, an ISS report mentioned the few months required to upgrade the diagnostic capability, the experience of the first months in identifying the most effective therapeutic schemes and the remediation to the early lack of personal protective equipment and medical equipment [10].

In this paper, we used publicly available resources to explore what circumstances and environmental conditions might have contributed to the variable time and space coordinates of the second epidemic wave in respect to the first before the start of the vaccination campaign in January 2021. This analysis might provide clues on how to be prepared against upcoming pandemics.

## Methods

We compared the COVID-19 CI, the death rate (DR), CFR, basic reproduction number (*R*_0_) and doubling time (DT) in the first *vs*. the second 2020 COVID-19 epidemic wave over the 20 Italian regions. We also investigated the association of those outcomes during the second wave with socio-economic conditions, environmental and lifestyle variables to explore any changes relative to previous similar analyses in the first wave [11]. For each region, we abstracted the daily incident cases of and deaths from COVID-19 from the Italian Ministry of Health website (http://ministerodellasalute.it) from the date of the first diagnosis up to 29 December 2020, the onset of the second wave declining phase. Household size, average *per capita* income, deprivation index, the proportion of the resident population by education level and resident population as of 1 January 2020 at the regional level were available from ISTAT (http://istat.it, and https://www.istat.it/it/files/2017/12/C07.pdf). We retrieved the circulating vehicles per 100 residents from https://www.comuni-italiani.it/statistiche/; the average March and November temperatures in the region capital, the two months of exponential increase of the epidemic curve, were retrieved from https://www.ilmeteo.it/portale/archivio-meteo/, and the regional population density from https://www.tuttitalia.it/regioni/densita/. Influenza vaccination rates were available by region and gender at https://www.epicentro.iss.it/influenza/coperture-vaccinali, for subjects aged ≥65, consistent with the World Health Organization (WHO) [12] and the US Centers for Disease Control and Prevention (CDC) [13] recommendations on influenza vaccination.

At the regional level, only the crude COVID-19 CFR (deaths/100 diagnoses) was available. However, as 94% COVID-19 deaths occurred among subjects aged ≥65 years, we considered the crude CFR to represent the mortality experience among the COVID-19 patients aged ≥65 years.

For each Italian region, we calculated the COVID-19 annual CI and DR in the first and in the second wave as it follows:
1
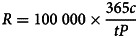

where *R* is the rate (CI or DR), *c* is the number of events (incident cases or deaths) occurring during the *t* time interval (corresponding to days from first diagnosis to 13 May 2020 in the first wave, and to days between 13 September and 29 December 2020, assumed as the starting and ending dates of the second wave), and *P* is the total resident population as of 1 January 2020. Rates were normalised to one year (365 days) to account for the inter-regional variation in the occurrence of the first case of COVID-19, and therefore varying *t* intervals during the first wave.

The reproduction number (*R*_0_) indicates the transmissibility of an uncontrolled infectious disease. We calculated it for each region at the beginning of the exponential growth of both the epidemic waves, starting from the first two consecutive days of doubling the cases up to the 30th day, as it follows [14]:
2
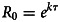

where *τ* is the median serial interval, i.e. the time between two successive cases in a chain of transmission of a disease (4.6 days in the case of COVID-19) [15], and *k* =  Δ*LN*(*N*)/Δ*T*, where the numerator is the difference between the natural log of the number of cases at *T*_0_ and *T*_1_, respectively, and Δ*T* = *T*_1_ − *T*_0_.

DT indicates the sequence of intervals at which a transmissible disease doubles its cumulative incidence [16]. It can be calculated as:
3
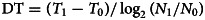

where *N*_0_ and *N*_1_ are the number of cases at the initial time *T*_0_ and the final time *T*_1_, respectively. In the exponential phase of the epidemic, it corresponds to the *k* value in equation [1], and it is an inverse measure of the rapidity of a pathogen's spread through a population, with immediate Public Health implications [17, 18]. For each region, we calculated DT every week during the first and second wave. Then, for each region, we plotted the two DT curves against weeks from the first case or from 13 September, respectively. We used the analysis of covariance to test the difference between the slope of the two curves. We empirically set at 50 days the doubling time of the transmission indicating its slowing down, i.e. the end of the logarithmic phase of the increasing incidence, and we calculated the difference in the number of weeks to reach DT = 50 (*δ*DT_50_) in the second respect to the first epidemic wave. The 50-days threshold was visually identified on the doubling time plots (Supplementary Fig. S1) as more clearly defining the divergence between the two curves than shorter or longer periods. *δ*DT_50_ would represent the change in the duration of the logarithmic spread of the disease: the larger the *δ*DT_50_, the longer the transmission rate took to slow down during the second epidemic wave; on the contrary, small values would indicate an approximately similar transmission rate between the two epidemic waves. We also explored the difference between the speed of spread in the two COVID-19 epidemic waves with 20-, 30- or 40-days thresholds. Results were equivalent using any of these alternative thresholds. As the 50-days threshold better discriminated the regions from each other, the results of the analyses with *δ*DT_50_ are presented throughout the paper.

We then explored whether the COVID-19 CI and CFR varied uniformly by region, first by ranking them by CI and CFR, and then by calculating the correlation between CI and CFR rank orders in the first *vs*. the second wave using the Spearman's correlation coefficient. Consistently with the analysis we conducted on the first COVID-19 epidemic wave [11] and to explore any differences between the two waves, we used multiple regression analysis to predict COVID-19 CI, COVID-19 DR, CFR in the second wave and *δ*DT_50_ as a function of average regional covariates. These included household size, *per capita* income, deprivation index, education level, circulating vehicles per 100 residents, average November temperature and rainfall in the region capital, population density, the proportion of the resident population aged ≥60 years and seasonal influenza vaccination rate among subjects aged ≥65 years. The deprivation index combines educational level, per cent of unemployed, housing and family conditions, to express the level of social disadvantage in a population [19]. Following a stepwise backwards procedure, the final model for each outcome only retained the covariates that improved the model fitness, as indicated by *a* > 5% increase in the *R*^2^ value. All the analyses were conducted with SPSS^®^ 20.0.

This study was conducted on publicly available data. No human subject was involved, and therefore it did not require ethical approval.

## Results

### Cumulative incidence, mass testing and social distancing in the first and the second epidemic waves

In the first wave, the nationwide, age-standardised COVID-19 CI was 17.0 per 1000 among males and 16.8 per 1000 among females and it increased to 106.8 among males and 105.7 among females in the second wave (males: *P* < 0.001; females: *P* < 0.001). The change in the age-standardised CFR was in the opposite direction: it was 10.7% in both males and females in the first wave, and 3.0% among males and 2.2% among females in the second wave (males: *P* = 0.04; females: *P* = 0.02).

**Fig. 1.**
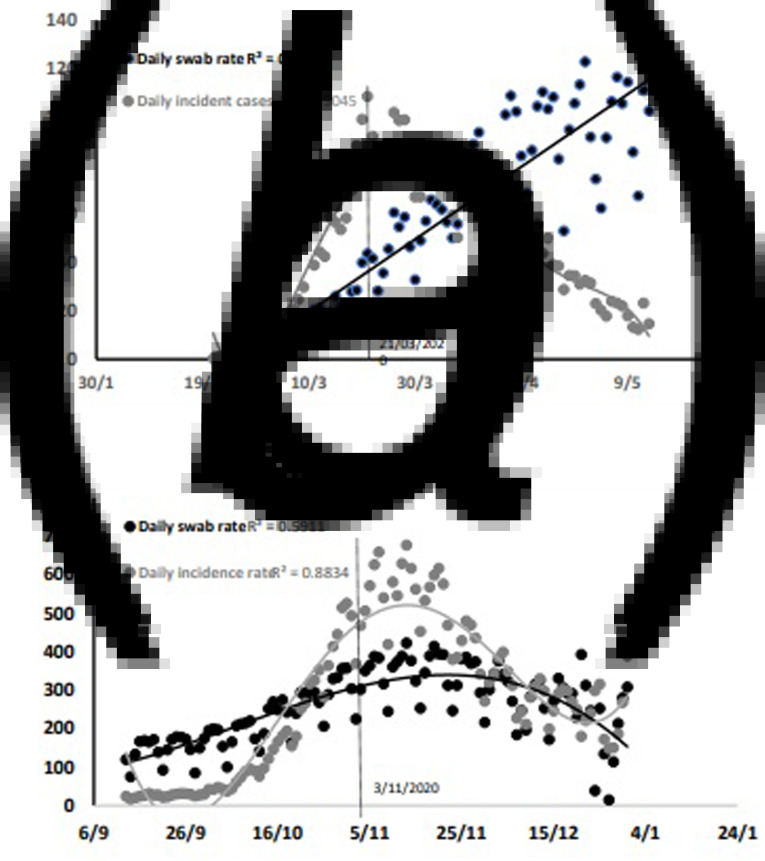
Daily COVID-19 incidence rate (per 1 000 000) (grey dots) and daily rate of nasopharyngeal swabs (per 100 000) during the first epidemic wave (21 February–13 May) in Italy (a) and during the second wave (b). While the scales differ by a factor of 10, the two graphs overlap for easier reading.

To explore whether the daily incident cases during the second wave were related to increasing detection through mass testing, we plotted the daily incident cases of COVID-19 (per 1 000 000) and the daily rate of nasopharyngeal swabs (testing rate, per 100 000) against time in the first (Fig. 1a) and the second wave (Fig. 1b). Note that the scales differ by a 10-times factor, while, in the graphs, they appear to overlap for easier reading. The supply of nasopharyngeal swabs matched the request a few weeks after the epidemic started. Therefore, there was a time interval between the increasing testing curve, which was best described by a linear regression (*R*^2^ = 0.815), and the epidemic curve, which best fit a five-level polynomial regression (*R*^2^ = 0.905), based on an increase in the *R*^2^ value >5%. Following a nationwide, generalised lockdown on 21 March, the epidemic curve and the testing rate curve started diverging, with mass testing reaching the top when the epidemic curve was at the bottom. During the second wave, both curves best fit a polynomial regression (testing curve: three-level, *R*^2^ = 0.5911; epidemic curve: four-level, *R*^2^ = 0.8834). The logarithmic increase of the incidence curve was not anticipated nor paralleled by a similar increase in the testing rate. At the top of the second wave, the testing rate had doubled, while the daily COVID-19 CI had increased seven times; both curves gradually declined afterwards.

### Case fatality rate in the first *vs*. the second epidemic wave

Having experienced a steeper increase in deaths during the first wave might have promoted more cautious behaviours during the second. To test the hypothesis, we first calculated the weekly deaths in the 10 regions with a first-wave CFR above the median, and in the 10 regions with a first-wave CFR below the median. We then calculated the ratio between the average weekly deaths in the second and in the first wave in each of these two groups of regions. Such ratio was 1.59 (standard deviation (s.d.) 0.73) in the regions which first-wave CFR was below the median, and 0.89 (s.d. 0.90) where it was above the median (*P* = 0.07).

To explore whether the prescribed measures were effective in mitigating the second epidemic wave, we also compared the average ratio between the weekly deaths during the second *vs*. the first wave in the regions with more restrictive (orange and red zones) *vs*. those with less restrictive measures (yellow zones). We did not observe a difference in COVID-19 deaths associated with more restrictive measures.

### Inter-regional variation and changes in cumulative incidence, and case fatality rate between the two 2020 COVID-19 epidemic waves

Table 1 shows the crude COVID-19 CI and CFR by region in Italy, along with the respective rank in the first and second waves. For both indicators, there was a good to moderate rank correlation between the two waves (CI: Spearman's correlation = 0.81, *P* < 0.001; CFR: Spearman's correlation = 0.54, *P* = 0.01, respectively), suggesting that the geographic distribution of the disease across Italy remained substantially unchanged during the second wave. We assumed the difference between the first wave CI ranking and CFR ranking (Δ*_r_*) to express the level of preparedness of the regional health system on facing the epidemic emergency. In the first wave, the average Δ*_r_* was 0 (s.d. 7.69), with the 10th and the 90th percentile of −9 and +10, respectively. Two regions, Veneto and Trentino Alto Adige, showed a Δ*_r_* lower than −9, i.e. had a high incidence and a low CFR; both are in north-eastern Italy. The Δ*_r_* was above or equal to +10 for Campania, Apulia and Sicily, indicating a low incidence and a high CFR; these are all in southern Italy. Such pattern was confirmed during the second wave (average Δ*_r_* = 0, s.d. 9.03), with Friuli Venezia Giulia (also in north-eastern Italy), joining Veneto and Trentino Alto Adige as the best performing regional health systems, and Abruzzo, Molise (also in southern Italy) and Sardinia (the second largest Italian island) joining the worst-performing regional health systems. The second wave rank difference greatly improved in Campania from 14 to 0. Molise and Sardinia, instead, went in the opposite direction, from a rank difference of −7 to +10, and from +2 to +12, respectively. Surprisingly, in the region most severely hit by the first epidemic wave, Lombardy, the two ranks were close to 0 in both COVID-19 waves.

**Table 1.** COVID-19 cumulative incidence (per 10 000) on annual base and case fatality rate by region in Italy in the first and in the second wave of the 2020 COVID-19 epidemic

	Cumulative incidence				Case fatality rate			
Region	First wave	Rank	Second wave	Rank	First wave	Rank	Second wave	Rank
Abruzzo	113.0	11	386.1	13	7.33	2	3.1	2
Basilicata	35.0	18	292.6	17	5.19	13	1.61	20
Calabria	28.1	20	181.1	20	5.34	12	2.26	11
Campania	37.8	17	507.2	8	7.31	3	2.53	8
Emilia Romagna	272.6	5	499.0	9	6.72	4	2.23	12
Friuli Ven. Giulia	128.1	9	609.5	5	4.61	16	1.92	19
Latium	54.2	14	412.9	12	5.47	11	2.44	9
Liguria	266.1	6	508.1	7	6.45	6	2.3	10
Lombardy	364.1	2	607.9	6	7.65	1	2.75	4
Marche	202.1	7	355.4	14	6.27	8	2	16
Molise	66.5	13	317.5	16	2.84	20	2.66	6
Piedmont	296.9	3	623.0	4	5.69	9	2.16	13
Apulia	50.5	15	334.0	15	6.48	5	3	3
Sardinia	41.6	16	277.8	19	4.97	14	2.54	7
Sicily	31.0	19	284.3	18	6.27	7	3.27	1
Tuscany	121.9	10	472.5	11	4.67	15	1.92	18
Trentino A. Adige	296.7	4	644.2	3	4.43	17	1.96	17
Umbria	79.4	12	491.9	10	3.32	19	2.04	15
Aosta Valley	482.6	1	784.1	1	5.67	10	2.75	5
Veneto	168.6	8	745.4	2	4.23	18	2.05	14
Spearman's correlation = 0.812, *P* < 0.001					Spearman's correlation = 0.543, *P* = 0.01			

Spearman's correlation.

### Speed of transmission by region in the first *vs*. the second epidemic wave

Table 2 shows the *R*_0_ and doubling time epidemic parameters in the first and the second wave of the 2020 COVID-19 epidemic by region. The *R*_0_ value ranged 1.81–4.74 across the 20 Italian region during the first wave (mean = 3.27, 95% CI 2.97–3.57), and it ranged 1.69–4.14 during the second wave (mean = 3.11, 95% CI 2.63–3.49) (*P* = 0.50). The lack of variation in the *R*_0_ would indicate that non-viral, local circumstances might have contributed to the inter-regional and inter-wave variations in the speed of transmission of the epidemic.

**Table 2.** Basic reproduction number (*R*_0_) and doubling time in the 2020 first and second epidemic wave of COVID-19 in the 20 Italian regions

	Reproduction number		Weeks at reaching DT_50_			Analysis of covariance (*P* value)
Region	First wave	Second wave	First wave	Second wave	*δ*DT_50_	
Abruzzo	3.60	1.73	10	13	3	0.07
Basilicata	3.43	1.84	7	13	6	0.02
Calabria	4.38	1.83	8	13	5	0.005
Campania	3.64	4.12	9	12	3	0.02
Emilia Romagna	2.85	3.65	10	13	3	0.04
Friuli Ven. Giulia	2.76	3.06	9	15	6	0.004
Latium	3.37	3.91	10	12	2	0.09
Liguria	2.95	3.52	11	11	0	0.75
Lombardy	4.74	4.14	10	12	2	0.54
Marche	3.24	2.93	9	13	4	0.003
Molise	1.81	1.69	8	13	5	0.08
Piedmont	3.21	3.72	11	12	1	0.67
Apulia	3.47	3.46	10	15	5	0.01
Sardinia	2.79	3.28	8	13	5	0.004
Sicily	2.08	3.62	10	13	3	0.01
Tuscany	3.38	3.75	10	11	1	0.09
Trentino A. Adige	4.03	3.21	10	12	2	0.04
Umbria	2.81	3.05	7	11	4	<0.001
Aosta Valley	3.14	1.77	9	11	2	0.01
Veneto	3.65	3.90	10	16	6	0.02
Average	3.27	3.11	9.3	12.7	3.4	
95% confidence interval	2.97–3.57	2.63–3.49	8.79–9.81	12.10–13.30	2.60–4.20	
*t* test (*P*)	−0.50		<0.001			

Table 2 also shows the number of weeks required to reach the doubling time threshold of 50 days, taken as the end of the increasing spread of the epidemic, by region and well as their inter-wave difference (*δ*DT_50_). The results indicate that, in the second wave, the transmission rate took longer (*δ*DT_50_ ≥ 3 weeks) to reach the 50-days threshold (*P* < 0.001) in 13/20 regions, resulting in a more severe burden to the health system and the economy. Among the seven regions in which *δ*DT_50_ was ≤2 weeks, five had experienced a first-wave CI and CFR above the median, and two below the median. A sensitivity analysis, setting a 20-, 30- or 40-days doubling time, confirmed that the weeks to reach the threshold were always significantly more in the second wave; 6/7, 5/6 and 4/7 regions with the respective *δ*DT ≤ 2 weeks had a first-wave CI and CFR above the median.

Table 2 also shows the results of comparing the slopes of the two DT curves. Supplementary Figure S1 shows the graphs describing the weekly changes of DT in the two epidemic phases for each region.

### The contribution of environmental, socio-economic and lifestyle factors to COVID-19 cumulative incidence, case-fatality rate and delayed transmission slowdown in the second epidemic wave

Table 3 shows the results of the multiple regression models predicting CI, death rate and CFR in the second wave, and *δ*DT_50_ as a function of environmental, socio-economic and lifestyle variables. The fraction of the resident population aged ≥60, the average November rainfall and the deprivation index did not substantially contribute and were, therefore, excluded. The results suggest that, in the second wave of the COVID-19 epidemic, the population density contributed to the increasing CI (*P* = 0.003) and death rate (*P* = 0.009); the number of circulating vehicles, taken as a surrogate for environmental particulate and gaseous emissions, significantly contributed to COVID-19 mortality (*P* = 0.003), whilst household size (*P* = 0.01) and an elevated average November temperature (*P* = 0.049) were inversely related. Household size was also inversely related to CFR (*P* = 0.01). The prolonged logarithmic increase of the COVID-19 transmission, represented by the *δ*DT_50_ value, was inversely related to the average regional educational level (*P* = 0.04). Assuming that a higher transmission rate would be due to lesser compliance with social distancing and the use of facial masks, this would have occurred in the regions with a lower education level. The *δ*DT_50_ was also directly related to the vaccination rate against seasonal influenza (*P* = 0.02), suggesting that its possible protective effect, observed during the first wave, if not due to chance, had dried up at the time of the onset of the second wave.

**Table 3.** COVID-19 cumulative incidence (on annual base), death rate (on annual base), case fatality ratio and delay in slowing down (*δ*DT_50_) the transmission of the disease in the second wave epidemic with respect to the first in the 20 Italian regions, as a function of environmental, socio-economic and lifestyle covariates

	CI Sep–Dec 2020 (per 1000)			Death rate Sep–Dec 2020 (per 10 000)			CFR Sep–Dec 2020 (per 100)			*δ*DT_50_ (days)		
Variables	Regression coefficient	Standard error	*P* value	Regression coefficient	Standard error	*P* value	Regression coefficient	Standard error	*P* value	Regression coefficient	Standard error	*P* value
Household size	–	–	–	−8.774	3.246	0.010	−10.884	4.213	0.014	−2.209	1.581	0.150
Average income (×1000 €)	1.446	0.625	0.027	–	–	–	−0.413	0.306	0.160	–	–	–
Vehicles (/100 residents)	0.359	0.239	0.129	0.257	0.082	0.003	0.148	0.103	0.142	−0.010	0.030	0.377
Average temperature	−1.891	0.988	0.064	−0.582	0.284	0.049	−0.426	0.458	0.259	–	–	–
Population density	0.063	0.020	0.003	0.022	0.008	0.009	–	–	–	–	–	–
Education	−0.881	0.518	0.094	−0.243	0.179	0.159	−0.094	0.239	0.369	−0.217	0.103	0.043
Vaccination rate (age >65)	–	–	–	–	–	–	–	–	–	0.174	0.070	0.018
	*R*^2^ = 0.810			*R*^2^ = 0.823			*R*^2^ = 0.607			*R*^2^ = 0.389		

## Discussion

Our results confirm previous reports of a diverging behaviour of the CI and CFR in the two 2020 waves of the COVID-19 epidemic in Italy [4] and provide some clues about the observed inter-regional variation. As an explanation for the first, a change in the prevalent SARS-CoV-2 variant between the two waves is unlikely, as the second wave features (no change in the basic reproduction number and a lower CFR in respect to the first wave) would be at odds with those reported for the *α* variant (increased transmissibility and increase in mortality) [5, 7, 8]. The effectiveness of the measures adopted in anticipation of a second wave, such as hiring more medical staff and opening dedicated hospitals, might explain why in some regions the CI/CFR ranking ratio improved or remained stable. The extreme changes observed in Campania, Molise and Sardinia would suggest that in the first region, but not in the second and third, adequate measures were taken to upgrade the preparedness of the local health system in the case of a second wave. Based on our findings, apart from possible, undetected changes in the prevalent SARS-CoV-2 variant, contributing factors would include (1) the early adoption and respect of social distancing measures in the first wave; (2) more effective therapies developed as a result of the medical experience accumulated during the first wave; (3) the increased availability of personal protective equipment, such as facial masks and hand sanitisers, which were insufficient during the first wave, and medical equipment, such as ventilators. The increase in supply became decisive in reducing the contagion among the medical staff and the general population later during the year, while ventilators contributed to reducing deaths; and (4) a more cautious attitude towards the epidemic in the regions that experienced higher mortality rates during the first wave, which might have lifted the level of acceptance of the social distancing measures. This hypothesis, in our view, appears more plausible than that of the herd immunity achieved far below the expected threshold to confer it, as others suggested [4]. As it concerns the beneficial effect of social distancing, our observations confirm a previous report [20].

Our analysis showed that, in some regions, the duration of the logarithmic increase of the epidemic curve did not vary between the two waves. In the rest of the regions, a significantly delayed DT50 and a less favourable CI/CFR ranking ratio characterised the second wave. Inter-regional variation in the preparedness and functioning of the local health system might have contributed.

Environmental factors, such as vehicular traffic emissions, average temperature, population density, average income, average educational level and average household size, did contribute to one or the other aspect of the COVID-19 epidemic in each region. We showed that, in the first COVID-19 epidemic wave, the vaccination against seasonal influenza provided some defence [11]. Such a finding was subsequently confirmed [21]. However, the influenza vaccination in the winter 2019–2020 did not beneficially affect the second COVID-19 epidemic wave. We cannot tell whether we observed a direct effect of the influenza vaccine limited to the first months after the vaccination or simply reflected less cautious attitudes in the second wave.

The finding of an inverse correlation between average household size and CI and CFR was counterintuitive. A possible explanation would be that living in a family would reduce participation in social life events, and that family responsibility and reciprocal watching in a household were effective in seeking medical advice earlier in the evolution of the disease.

Weather conditions, such as humidity, wind speed and atmospheric pressure, might also modulate the spread and lethality of COVID-19 [22]. In our previous report, the average March temperature did not impact on the COVID-19 outcomes [18]. Instead, in the present study, the average November temperature was inversely related to COVID-19 mortality, while the average precipitation was not.

An ISS report described a decrease in the age- and gender-standardised case-fatality rate during the second wave and called for an effect of improved diagnostic capability [10]. We acknowledge the unavailability of age- and gender-standardised events at the regional level as a limitation. Investigating whether the decrease in the number of cases among the elderly, which was a feature of the second epidemic wave, was consistent or not by region was therefore unfeasible [23]. Besides, our results show that, while the issuing of social distancing measures effectively bent the epidemic curve, the testing rate followed rather than anticipated the evolution of the epidemic curve. This would suggest that mass testing contributed negligibly in early detecting incident cases and preventing the second wave.

A statement recurrent among statisticians says: ‘correlation does not imply causation’. Ours was a hypothesis-generating ecological study, in line with the epidemiological study design that prevailed in this first stage of inquiry into the contributing factors in the spread of the COVID-19 pandemic in the absence of individual data [24]. We did observe correlations. Therefore, interpreting our findings exposes to the so-called ecological fallacy, as the whole regional populations and not the individuals were the exposed unit [25, 26]. For instance, the association we observed with vehicular traffic and average temperature might be explained with a greater probability of social contacts, rather than particulate emissions or prolonged viral survival in the environment.

Further limitations include the availability of influenza vaccination rates only for age 65 years or older, as prescribed by WHO and CDC [11, 12]. About 96% of deaths from COVID-19 occurred among subjects ≥60 years old, while the same age group accounted for 54% of incident cases during the first wave *vs*. 33% in the second wave. Such change might alone explain the reduction in CFR during the second wave, as the most susceptible elderly and those with health conditions were more likely to have died during the first wave, the so-called harvest effect [27]. On the other hand, the proportion of residents aged ≥60 years ranges from 25.6 to 35.7 across regions. Therefore, the regional fraction of the resident population aged ≥60 years was not predictive of incidence and mortality from COVID-19 (CI: *P* = 0.79; DR: *P* = 0.30) and, in our opinion, comparing crude CI and DR across regions would still provide valuable results.

Also, the evolution of the infection and the control measures changed rapidly. For this reason, we restricted our analysis to the period before vaccines became available and considered the effect of the nationwide control measures on the epidemic curve.

We used the rate of circulating vehicles as a surrogate for exposure to environmental pollutants, as we did not have access to environmental monitoring data. Still, our observation of a direct correlation with COVID-19 DR and *δ*DT_50_ corroborates the hypothesis of increased susceptibility to/severity of COVID-19 due to chronic exposure to atmospheric pollutants causing lung damage and higher vulnerability to virus entrance and replication [18, 28, 29]. However, uncertainty remains due to our ecological study design. Further studies with individual measurements of exposure are warranted to confirm the hypothesis.

## Conclusion

As similar events are part of the history of humanity, it is predictable that pandemics might be part of our future as well. The evolution of the 2020 COVID-19 epidemic in Italy indicates that a forward-looking public health preparedness is warranted in case of emerging transmissible diseases against which immunity is lacking.

## Data Availability

The data underlying this article will be shared on reasonable request to the corresponding author.

## Supplementary material

For supplementary material accompanying this paper visit https://doi.org/10.1017/S095026882200084X. click here to view supplementary material
