# Supplementary material for: The determinants of the changing speed of spread of COVID-19 across Italy
Source: Epidemiol Infect. 2022 May 6;150:e94. doi: 10.1017/S095026882200084X (PMC9114753; doi:10.1017/S095026882200084X)
Supplement: Supplementary file 1 [file S095026882200084Xsup001.docx]

Supplementary Figure 1.

Weekly doubling time curves in the first and the second COVID-19 epidemic wave by region in Italy (F test from analysis of covariance).

Analysis of covariance: F = 3.69; *p* = 0.066

Analysis of covariance: F = 6.75; *p* = 0.016

Analysis of covariance: F = 9.40; *p* = 0.005

Analysis of covariance: F = 6.28; *p* = 0.019

Analysis of covariance: F = 4.66; *p* = 0.040

Analysis of covariance: F = 10.34; *p* = 0.004

Supplementary Figure 1 (continues)

Analysis of covariance: F = 3.07; *p* = 0.092

Analysis of covariance: F = 0.1; *p* = 0.754

Analysis of covariance: F = 0.38; *p* = 0.543

Analysis of covariance: F = 10.58; *p* = 0.003

Analysis of covariance: F = 3.27; *p* = 0.083

Analysis of covariance: F = 0.19; *p* = 0.667

Analysis of covariance: F = 7.52; *p* = 0.011

Sardinia: F = 10.06; *p* = 0.004

Supplementary Figure 1 (continues)

Analysis of covariance: F = 7.42; *p* = 0.011

Analysis of covariance: F = 3.17; *p* = 0.087

Analysis of covariance: F = 4.32; *p* = 0.037

Analysis of covariance: F = 19.04; *p* = 0.0002

Analysis of covariance: F = 6.92; *p* = 0.015

Analysis of covariance: F = 5.67; *p* = 0.025
